# A Study of Combining Elastin in the Chitosan Electrospinning to Increase the Mechanical Strength and Bioactivity

**DOI:** 10.3390/md19030169

**Published:** 2021-03-22

**Authors:** Hengjie Su, Tomoko Fujiwara, Joel D. Bumgardner

**Affiliations:** 1 Department of Biomedical Engineering, UT-UofM Joint Graduate Program in Biomedical Engineering, The University of Memphis, Engineering Technology Bldg #330, Memphis, TN 38152, USA; suhengjie@hotmail.com; 2 Department of Chemistry, The University of Memphis, Smith Hall #409, Memphis, TN 38152, USA; tfjiwara@memphis.edu

**Keywords:** electrospinning, chitosan, elastin, mechanical strength

## Abstract

While electrospun chitosan membranes modified to retain nanofibrous morphology have shown promise for use in guided bone regeneration applications in in vitro and in vivo studies, their mechanical tear strengths are lower than commercial collagen membranes. Elastin, a natural component of the extracellular matrix, is a protein with extensive elastic property. This work examined the incorporation of elastin into electrospun chitosan membranes to improve their mechanical tear strengths and to further mimic the native extracellular composition for guided bone regeneration (GBR) applications. In this work, hydrolyzed elastin (ES12, Elastin Products Company, USA) was added to a chitosan spinning solution from 0 to 4 wt% of chitosan. The chitosan–elastin (CE) membranes were examined for fiber morphology using SEM, hydrophobicity using water contact angle measurements, the mechanical tear strength under simulated surgical tacking, and compositions using Fourier-transform infrared spectroscopy (FTIR) and post-spinning protein extraction. In vitro experiments were conducted to evaluate the degradation in a lysozyme solution based on the mass loss and growth of fibroblastic cells. Chitosan membranes with elastin showed significantly thicker fiber diameters, lower water contact angles, up to 33% faster degradation rates, and up to seven times higher mechanical strengths than the chitosan membrane. The FTIR spectra showed stronger amide peaks at 1535 cm^−1^ and 1655 cm^−1^ in membranes with higher concentrated elastin, indicating the incorporation of elastin into electrospun fibers. The bicinchoninic acid (BCA) assay demonstrated an increase in protein concentration in proportion to the amount of elastin added to the CE membranes. In addition, all the CE membranes showed in vitro biocompatibility with the fibroblasts.

## 1. Introduction

Guided bone regeneration (GBR) membranes are widely used to help regenerating bone tissue in cranio-maxillofacial, periodontal, implant treatments, etc. [1,2,3]. In bone regeneration treatment, GBR membranes provided a barrier function to prevent premature bone graft resorption and increase the amount of new bone regeneration. Several studies and reviews have highlighted the effectiveness of GBR membranes in regenerating bone [2,3,4]. GBR membranes are divided into nonabsorbable membranes and degradable membranes. Since nonabsorbable membranes require second surgery for extraction, degradable membranes are generally preferred. Commercial degradable GBR membranes are made of collagen (BioMend Extend, Bio-Gide) and poly-lactic acid (GUIDOR), etc. However, they have several shortcomings, such as weak mechanical handling properties and the unpredictable degradation of collagen membranes [5,6,7].

Chitosan has attracted attention for its use as a GBR membrane, because it is nontoxic, biocompatible, biodegradable, and has a low cost. Electrospun chitosan membranes are a focus of much research and development for GBR applications due to their biocompatibility, biodegradability, support function for newly formed bone, and nanofiber-porous structure that mimics the nanofiber structure of the native extracellular matrix that is also cell-occlusive [8,9,10,11].

The mechanical tear strength is an important property in evaluating the membrane handling ability, since membranes need to be strong enough to be manipulated and secured in place during implantation operations [4]. Thicker GBR membranes with a stronger mechanical strength have been preferred for GBR treatments but may lead to the dehiscence of soft tissues and membrane exposure [4]. In our previous study, the tear strength of electrospun chitosan membranes was similar to the BioMend Extend collagen membrane [1]. However, the electrospun chitosan membranes were still weaker than the Bio-Gide collagen membrane (Geistlich Pharma AG) that is preferred by dentists because of its strong handling ability [1].

To increase the tear strength, elastin may be incorporated into the electrospun chitosan membrane. Elastin is an extracellular protein that provides the elasticity of tissues, such as blood vessels, ligaments, the lungs, and skin [12]. Researchers have explored using elastin in tissue-engineered skin [13], vascular grafts [14], heart valves [15], and cartilage [16] constructs. Collagen [14,17], poly-lactic-co-glycolic acid (PLGA) [18,19], and polycaprolactone (PCL) [20], etc. have been mixed with elastin to make electrospun materials. A study from Grover et al. showed that adding both insoluble and soluble elastin increased the tensile strength of a collagen porous scaffold [21]. Soluble elastin resulted in increased tensiles compared with insoluble elastin when added to collagen scaffolds [21]. In addition to its structure function, elastin can accelerate tissue regeneration and induce an osteogenic response [12]. Solubilized elastin has been reported to enhance the biocompatibility of synthetic materials [22,23].

In this study, the effects of incorporating a hydrolyzed soluble elastin from a bovine neck ligament into the properties of electrospun chitosan membranes were examined. The role of increasing the elastin composition was explored through evaluations of the fiber size and morphology, water contact angle, Fourier-transform infrared spectroscopy (FTIR), elastin incorporation, degradation, tear strength, and cell.

## 2. Materials and Methods

### 2.1. Membrane Preparation

Electrospun membranes were prepared based on a previously described method using a shrimp-derived chitosan with a degree of de-acetylation (DDA) = 71% and molecular weight = 311.5 kDa, as reported by the manufacturer (Primex EHF, Siglufjordur, Iceland) [1,7,9,11]. In brief, the chitosan solution was prepared by dissolving 5.5% (*w*/*v*) chitosan in 7:3 (*v*/*v*) trifluoracetic acid/dichloromethane solution overnight [1,7,9,11]. Before electrospinning, elastin (ES12, Elastin Products Company, Owensville, MO, USA) was added into the chitosan solution at 0%, 1%, 2%, and 4% (*w*/*v*) of the spinning solution. A 10-mL solution was prepared for each chitosan membrane with 0% and 1% elastin, and a 5-mL solution was prepared for each membrane with 2% and 4% elastin. A 10-mL syringe with an 18-gauge blunt needle was filled with the chitosan–elastin (CE) solution and then placed on a syringe pump set to 15 μL/min. An aluminum foil-covered plate 15 cm away from the 26-kV voltage needle tip was rotated to collect the electrospun nanofibers. Membranes were then subjected to post-spinning immersion in triethylamine (TEA)/di-tert-butyl decarbonate (tBOC), as previously described [1,11]. In this process, TEA is used to remove TFA salts that form on the chitosan polymer chains during the electrospinning process. Then, the reaction with tBOC is used to cap the chitosan free amine groups on the fiber surfaces to prevent fiber swelling in aqueous environments [1]. Briefly, membranes were immersed in 10% TEA/acetone for 48 h to remove TFA salts formed during the spinning process. Membranes were then submerged in 0.1-g/mL tBOC/acetone solution with stirring for 36 h, followed by rinsing with acetone, and dried between two pieces of nylon mesh in air.

### 2.2. Surface Morphology

The surface morphology of the CE membranes was observed by scanning electron microscopy (SEM) using an EVO HD15 (Carl Zeiss AG, Jena, Germany) scanning electron microscope. CE membranes were adhered to an SEM stub and coated with 5-nm gold palladium. Samples were observed from 2500× to 6500×. Fiber diameters were calculated from 20 randomly selected fibers from each membrane using SEM image analysis software. Triplicate samples of each membrane type were evaluated.

### 2.3. Water Contact Angle

The hydrophobic/hydrophilic property of the CE membranes was evaluated by the water contact angle. Contact angles of the water drop contacting the membrane surface were recorded by a VCA optima measurement machine (AST Products, Billerica, MA USA). There was one drop per specimen, and five samples of each membrane type were tested.

### 2.4. Fourier-Transform Infrared Spectroscopy (FTIR)

FTIR was used for evaluating the elastin content in the CE membranes. FTIR spectra were collected using a Nicolet 380 FTIR spectrometer (Thermo Fisher Scientific, Waltham MA, USA). Three samples of each TEA/tBOC-treated CE membrane were scanned from 500 cm^−1^ to 4000 cm^−1^ 32 times.

### 2.5. Bicinchoninic Acid (BCA) Assay

The BCA assay was used for evaluating the elastin concentration in the CE membranes. Each TEA-treated CE membrane was cut into 4-mg pieces (*n* = 3). These samples were dissolved in a solution containing 0.014% (*w*/*v*) sodium deoxycholate and 6.55% (*w*/*v*) trichloroacetic acid overnight. After centrifuging the solution at 5000 rpm for 15 min, the supernatant was carefully removed without disturbing the bottom pellet. A 5% sodium dodecyl sulfate (SDS) solution was added to dissolve the protein pellet. The sample solution was tested using the Pierce BCA Protein assay (Thermo Fisher Scientific, Waltham, MA, USA). A standard curve made from elastin solution was used for calculating the protein concentration.

### 2.6. Degradation

The degradation of the CE membranes was evaluated based on the mass loss. Samples of each CE membrane were cut into 3-cm^2^ squares (*n* = 3) for each time point of 1 week, 2 weeks, and 4 weeks. After recording the original sample weight, samples were soaked in phosphate-buffered saline (PBS) solution containing 100-μg/mL lysozyme, 500-I.U./mL penicillin, 500-μg/mL streptomycin, and 25-μg/mL amphotericin-B at 37 °C. The solution was changed every 2 days. At the 1-, 2-, and 4-week time points, the membranes were retrieved, rinsed in distilled-deionized water, dried for 48 h, and weighed (mg) to record the change in mass. Compared with the lysozyme level in human plasma, which is 3–8 μg/mL, a high level was used in the experiment to accelerate the degradation and magnify the potential differences over the course of the experiment.

### 2.7. Mechanical Strength

A mock surgical tack test was used to evaluate the mechanical tear properties of the TEA/tBOC-treated CE membranes as an indicator of the clinical handling ability. Specimens of 10 × 30 mm were tacked onto a 7.5 × 7.5 × 0.5-cm white oak board as a bone analog [1] using the AutoTac system kit, (Biohorizons, Birmingham, AL, USA). A point 5 mm from the wide top and 5 mm from the length edge of the specimens were used for tacking membranes onto the wood board. The wood was positioned in the lower clamp of an Instron^TM^ Model 4456 mechanical test frame, and the free end of the membrane was positioned in the upper clamp. The load cell used was 50 N, and the extension rate was 1 mm/min. The maximum load was recorded in Newton (N) and then normalized to the membrane thickness. The stretching lengths were recorded in millimeter (mm) and then normalized to the original length. Triplicate samples of each type of membrane were tested.

### 2.8. In Vitro Cell Proliferation

Ethylene oxide gas-sterilized disc-shaped chitosan membrane specimens (diameter = 1.5 cm) were inserted into 24-well plates for the evaluation of osteoblast and fibroblast growths on the membranes for 7 days. Membranes were rinsed in culture media overnight and then seeded with NIH 3T3 cells (ATCC, Manassas, VA, USA) at 5 × 10^4^ cells/well. Cells were grown in MEM-α medium mixed with 10% FBS and 500-I.U./mL penicillin, 500-μg/mL streptomycin, and 25-μg/mL amphotericin-B. Cell growth was measured using the Cell Titer Glo^TM^ luminescent cell viability assay (Promega, Madison, WI, USA). The assay was based on the luciferin luciferase reaction to measure the amount of ATP, which was proportional to the cell numbers (*n* = 4/sample per day per cell type). The data were reported in relative luminescent units (RLU).

### 2.9. Statistical Analysis

One-way analysis of variance (ANOVA) at the 0.05 level of significance was used for analyzing the results of the fiber diameter, water contact angle, protein concentration, and mechanical strength, with the membrane types as the factor. Two-way ANOVA at the 0.05 level was used for analyzing the degradation and cell proliferation results, using the membrane types as one factor and time points as another. Tukey’s post-hoc tests were used to distinguish significantly different groups.

## 3. Results and Discussion

### 3.1. Surface Morphology

**Figure 1:**
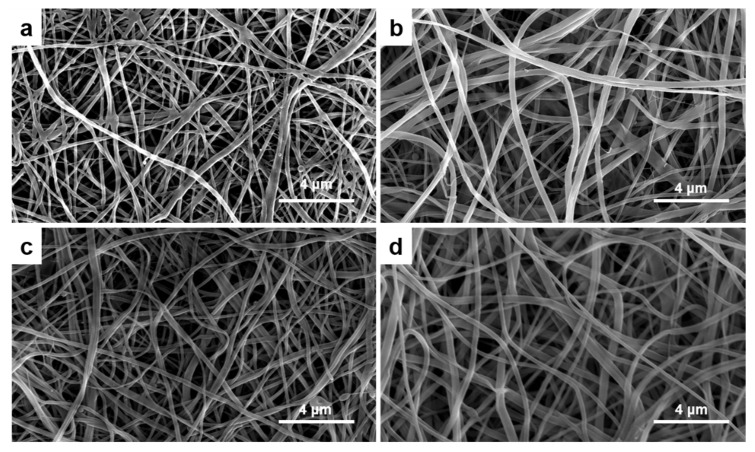
The SEM graphs of the (**a**) 0% elastin chitosan–elastin (CE) membrane, (**b**) 1% elastin CE membrane, (**c**) 2% elastin CE membrane, and (**d**) 4% elastin CE membrane with 6500× magnification. A fibrous structure could be observed in all the CE membranes.

**Figure 2:**
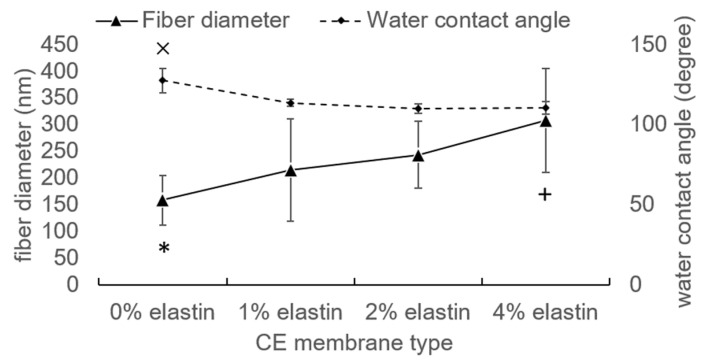
The fiber diameter and water contact angle of the CE membranes. ×, *, and + denoted the significant differences. Bars represent the mean ± standard deviation.

The CE membranes showed that they have well-preserved nanofibrous structures after TEA/tBOC treatment in the SEM graphs (Figure 1). The fiber diameters increased with the increasing percent of elastin (Figure 2). The fiber diameters of the no elastin and 1% CE membranes were significantly smaller than the 2% and 4% CE membranes, and the diameter of the 4% elastin CE membrane was significantly larger than the 0% and 1% elastin CE membranes but not different from the 2% CE membrane (*p* = 10^−4^).

In prior studies, it was demonstrated that the TEA/tBOC treatment prevented swelling and the loss of the nanofiber structure of electrospun chitosan membranes in aqueous solutions [1]. This same treatment also prevented the swelling and loss of the fiber structure of the CE electrospun membranes in aqueous solutions in this study. This was important to determine, since retention of the nanofiber structure is an important feature of these membranes for mimicking the native extracellular matrix, ECM, fibular structure, maintaining cell occlusivity while enabling the free diffusion of factors between gingival and bone compartments for GBR applications [4,7,8].

The chitosan nanofibers in this work exhibited fiber diameters on par with the previous work, which was 330 ± 130 nm [1]. The differences in the mean values between the two studies are likely related to the seasonal differences in the moisture and temperature at the time of spinning, since the spinning procedure during done at ambient atmospheric conditions, and changes in the temperature and humidity are known to have significant effects on the sizes of electrospun fibers [24,25]. Even though there was some variability in the fiber diameters between the studies, overall, the electrospinning process produced fibers in the nano-scale range, which was considered an advantage for mimicking the native ECM fiber network.

Other research groups electrospun elastin either by itself or when combined with collagen. Elastin fibers were electrospun with diameters between 1 μm to 500 nm, depending on the spinning conditions [14,26]. When combined with collagen, electrospun fibers with diameters between 490 nm to 800 nm were made [14,26]. It is interesting to note, though, that the diameters of the collagen–elastin composites were typically larger than their pure collagen control counterparts, suggesting that elastin tends to increase fiber diameters. This may be due to the non-crystalline network structure of the elastin protein [14,26]. Similar effects of increasing fiber size with increasing additions of elastin to the electrospun chitosan fibers were observed in this work.

### 3.2. Water Contact Angle

The hydrophobic/hydrophilic properties of the CE membranes were tested by the water contact angle. Chitosan membranes with 1–4% (*w*/*v*) elastin all had significantly reduced the water contact angles as compared to the 0% elastin control (*p* = 10^−4^; Figure 2). However, the water contact angles for all membranes were greater than 100°, indicating that the membranes still exhibited highly hydrophobic characteristics.

Compared with the previous study, the water contact angle of the chitosan membrane was 119 ± 14°, which was coincident with the results here [1]. The addition of an elastin protein to the spinning solution decreased the hydrophobic characteristic of the electrospun membranes. This increase in hydrophilicity is reasonable, because ES12 elastin is water-soluble. However, because prior research demonstrated that the tBOC modification results in three methyl groups being attached to the surface of the fibers [1], the membranes containing elastin remained largely hydrophobic. It is this hydrophobic character that enables the membranes to retain their nanofiber structure, as seen in this work.

### 3.3. FTIR

**Figure 3:**
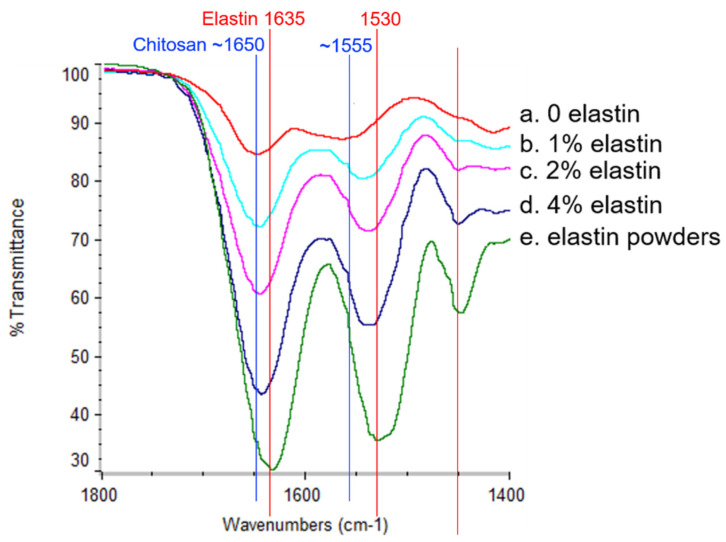
The Fourier-transform infrared (FTIR) spectra of the pure elastin powders and the CE membranes. The peaks at 1530 cm^−1^ and 1635 cm^−1^ corresponded to the elastin amide band II and amide band I (red lines indicated positions). The peaks around 1555 cm^−1^ and 1650 cm^−1^ corresponded to the chitosan amide band II and amide band I (blue lines indicated positions). From the top to the bottom at the two peak positions, the curves represented the (a) 0% elastin CE membrane, (b) 1% elastin CE membrane, (c) 2% elastin CE membrane, (d) 4% elastin CE membrane, and (e) the elastin powders, respectively.

The FTIR spectra of the CE membranes and elastin powder are shown in Figure 3. Elastin had two main peaks at around 1530 cm^−1^ and 1635 cm^−1^ that corresponded to the amide II and amide I bonds, respectively [27], and these peaks overlapped with the chitosan amide II and amide I peaks around 1555 cm^−1^ and 1650 cm^−1^, respectively [1,7]. Since the amide I and II peaks attributed to elastin added to the peaks of chitosan, as the elastin content increased, the intensity of both amide peaks also increased. Hence, the increase in the FTIR amide I and II peaks indicated an increasing elastin incorporation due to the increasing amount of elastin in the spinning solution. These results indicated that elastin was incorporated into the electrospun fibers.

### 3.4. Bicinchoninic Acid (BCA) Assay

**Figure 4:**
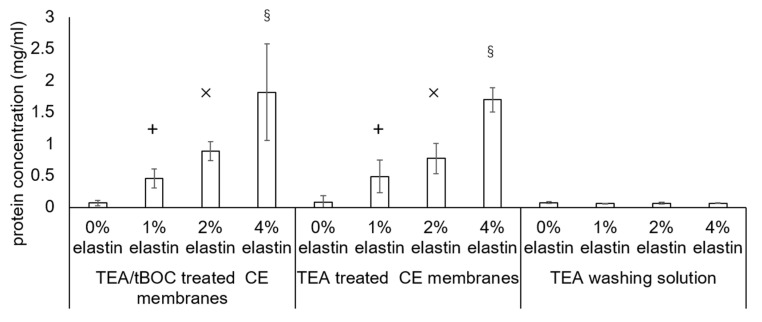
Bicinchoninic acid (BCA) assay result of the triethylamine (TEA)—di-tert-butyl decarbonate (tBOC)—treated chitosan-elastin (CE) membranes, TEA—treated CE membranes, and TEA washing solutions during the TEA treatment. The protein ratio significantly increased with the increased elastin concentrated CE membranes. There was no significant difference in between the two procedure-treated CE membranes. The protein ratios of the TEA washing solutions were extremely lower than the 1%, 2%, and 4% elastin CE membranes. +, ×, and § denoted the significant differences from the other groups (*p* < 0.05). Bars represent the mean ± standard deviation.

The results of the protein assay to determine the amount of elastin incorporated into the electrospun fibers is shown in Figure 4. Membranes subjected only to TEA (no tBOC reaction) were examined to see if the TEA/tBOC reaction process resulted in any loss of elastin from the membranes. In addition, the acetone wash solutions were also evaluated for any extracted elastin protein. The results showed that there was a significant increase in the measured protein contents of the fibers with an increasing percent of elastin (*p* = 10^−4^). It was further noted that there was no difference in the amount of proteins measured in the TEA or TEA/tBOC-treated samples, indicating that the tBOC treatment did not result in a significant loss in elastin proteins from the electrospun fibers during the reaction. The low protein content of the TEA washing solution further confirmed that the TEA/tBOC treatments were not extracting or washing out the elastin from the electrospun fibers.

While there was a proportional increase in the total protein in the fibers with increasing elastin additions to the spinning solution, the total protein measured was approximately one-sixth of the theoretical amount based on the original chitosan–elastin spinning solution mixture. However, the BCA assay may not have accurately measured the amount of elastin extracted from the electrospun fibers due to the interference of chitosan with the assay. To verify the interference, the pure elastin powders and chitosan–elastin powders mixed at the same weight ratios as used in the spinning process were tested using the BCA assay kit. The result showed that the BCA assay identified almost all the pure elastin powders but only identified around one-fifth of the elastin in the elastin–chitosan mixtures (data not shown). Hence, the BCA assay likely underestimated the amount of elastin in the fibers. In addition, it is also possible that the TFA might have degraded some elastin during the electrospinning process. Further studying will be needed to determine the exact amount of elastin in the fibers and/or if the TFA causes degradation of the elastin protein. Nevertheless, the data correlated qualitatively with the FTIR data showed that increasing amounts of elastin are being incorporated into the membranes with increasing additions of elastin to the spinning solution.

### 3.5. Degradation

**Figure 5:**
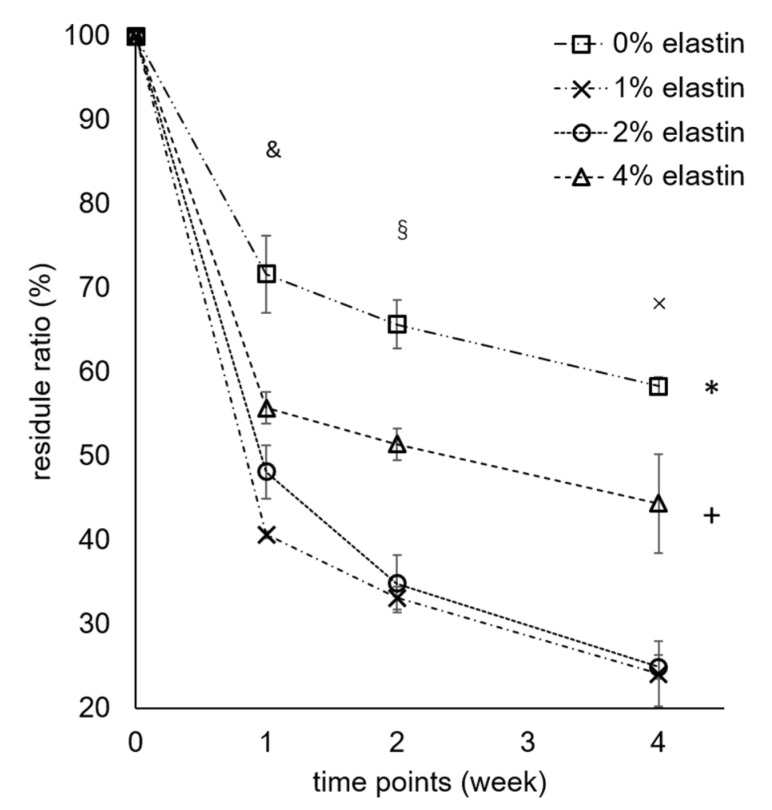
Degradation results of the CE membranes. Bars represent the mean ± standard deviation. * and + denote the significant differences from the 1% and 2% elastin CE membranes (*p* < 0.05). §, &, and × denote the significant differences from week 0 (*p* < 0.05).

The degradation results showed that the 1%, 2%, and 4% elastin CE membranes had higher degradation rates than the chitosan membrane. After four weeks, the chitosan membrane had a significantly greater residual mass than the 1%, 2%, and 4% CE membranes (*p* = 10^−4^; Figure 5). The 4% elastin CE membrane had a significantly higher residual mass than the 1% and 2% CE membranes and significant lower residual mass than the chitosan membrane (*p* = 10^−4^). The weight of all the membranes was significantly reduced over time from the initial week to four weeks.

All the membranes showed significant degradation after four weeks, which proved that the CE membranes were degradable (*p* = 10^−4^). In a previous study, the chitosan membrane significantly decreased in membrane weight after each time point [1]. After one week, two weeks, and four weeks, the membrane residue was 89% ± 6%, 65% ± 4%, and 56% ± 2%, respectively, in the previous study. Compared with this study, the chitosan membrane had 72% ± 5%, 66% ± 3%, and 58% ± 1% residual mass after one, two, and four weeks, respectively, which was consistent with our previous work [1]. After combining the elastin in the chitosan electrospun membranes, the degradation rate became higher than the chitosan membrane, which might be due to elastin causing a disruption in the chitosan crystalline structure, making the amorphous polymer more susceptible to degradation. The lysozyme concentration was very high, and so, these in vitro tests were not necessarily predictive of how membranes might degrade in vivo or whether they would meet the four-to-six-month maintenance to provide osteo-regeneration protection. The increase in degradation needs to be balanced with the need to provide a barrier function for the four to six months [1]. If the faster healing of tissues occurs with CE membranes, faster degradation may not be an issue. Further studies evaluating degradation in vivo over these clinically relevant time frames are needed to confirm these properties.

It was interesting that the 4% elastin had a slower degradation rate as compared to the 1% and 2% elastin membranes. The reason for this is not completely understood but may be related to the fact that elastin is not degradable by lysozyme and does not hydrolyze readily. Hence, membranes with a higher elastin content may show slower degradation in the lysozyme solution. This hypothesis will need to be further explored, as well as additional degradation studies involving both lysozyme and elastases, to better understand the degradation processes of these membranes.

### 3.6. Mechanical Strength

**Figure 6:**
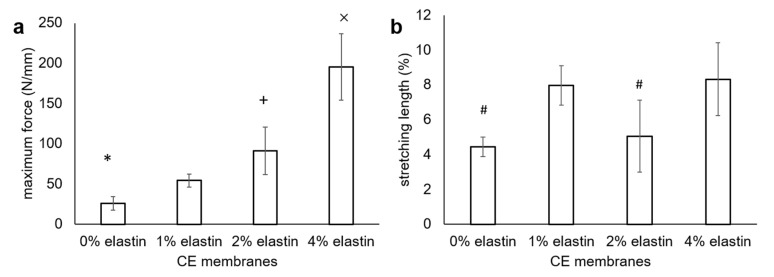
(**a**) Surgical tear strength and (**b**) stretching lengths of the CE membranes. The maximum load was recorded in Newtons (N) and then normalized to the membrane thickness. Bars represent the mean ± standard deviation. *, +, and × denote the significant differences from the 1% elastin CE membrane (*p* < 0.05). # denotes the significant differences from the 1% and 4% elastin CE membranes (*p* < 0.05).

The results of the surgical tac test showed an increased tearing strength with the increased elastin concentration (Figure 6). The tear strengths of the 0% to 4% elastin CE membranes increased from 26 ± 8 N/mm to 54 ± 8 N/mm, 91 ± 29 N/mm, and 195 ± 41 N/mm, respectively (Figure 6a). There were significant differences in between the chitosan membrane, 2% elastin membrane, and 4% elastin CE membrane (*p* = 0.04). The total elongation of the 0–4% CE membranes were 4% ± 1%, 8% ± 1%, 5% ± 2%, and 8% ± 2%, respectively (Figure 6b). The stretching lengths of the 0% and 2% elastin CE membrane groups were significantly shorter than the 1% and 4% elastin CE membrane groups.

Adding elastin in the electrospun chitosan membranes significantly improved the tensile properties over that of the plain electrospun chitosan membranes. The mechanical tear strengths of the 2% and 4% elastin CE membranes were similar to or greater than the tear strengths of the two commercial collagen membranes (Bio-Gide 134 ± 22 N/mm and BioMend Extend 55 ± 8 N/mm) tested under similar conditions [1]. It is noted too that these CE membranes were thinner than the commercial collagen membranes. The ability to achieve similar tear strengths with thinner membranes may be an advantage for handling and implanting in sites where gingival tissues may be thin, as well as providing improved protection to the underlying bone graft materials. In addition, the elongation percent of the CE membranes was generally increased over that of the chitosan membranes. This coupled with the increased tear strength would indicate that an elastin addition increased the toughness of the CE membranes. The highly elastic character of elastin increases the elasticity properties and, hence, increases the tearing strength of the CE membranes. Though the elongation was not in proportion to the elastin concentration as expected, this might be an artefact of having thinner samples for the 2% and 4% CE membranes than the 0% and 1% CE membranes. The change in sample sizes was an effort to conserve materials in order to be able to have sufficient samples for testing. The tacking and clamping of the samples in the test set-up may have caused more damage to the thin membranes than the thicker ones, thus reducing their elongation. This may be why the 2% elastin CE membrane showed less stretching length than the 1% elastin CE membrane. However, the effect of increasing the elongation from 2% to 4 % elastin in the CE membranes was the same as the increase from 0% to 1% elastin. The difference in the thicknesses of the test specimens was a limitation of this work, and future studies will be aimed at further evaluating their mechanical properties. In addition, it would be meaningful to also test CE membranes in wet/hydrated conditions in the future to gain a better understanding of their mechanical toughness under physiological conditions.

The tear strength of the chitosan membrane in this study was approximately 50% lower that the tear strength of the TEA/tBOC-modified membranes previously reported [1]. This difference is attributed to the differences in spinning from the previous study, when a 30-mL chitosan solution was used to prepare the electrospun chitosan membranes as compared on only a 10-mL chitosan solution used in this study. The chitosan membrane made from a 30-mL solution (thickness = 0.15 mm) was thicker than the membrane made from the 10-mL solution (thickness = 0.1 mm). From the rough evaluation, the triple volume solution and the more compact structure of the membranes in the previous study as compared to the membranes in this study may explain the higher tear strength of the chitosan membrane in the previous study.

### 3.7. In Vitro Cell Proliferation

**Figure 7:**
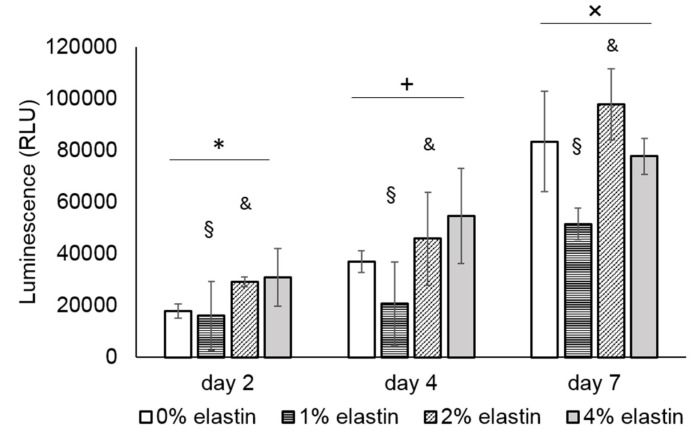
Graph shows the mean relative luminescent units (RLU) of the NIH 3T3 cell proliferation on the CE membranes. There was a significant difference in between the time points. *, +, and × denote the significant differences of each time point (*p* < 0.05). The cell proliferation on the 1% and 2% elastin membranes was significantly different from the other membranes (*p* = 10^−4^). § and & denote the significant difference of the 0% and 4% elastin CE membrane groups (*p* < 0.05). Bars represent the mean ± standard deviation.

The cell proliferation results are shown in Figure 7. The NIH 3T3 fibroblast cells showed significant proliferation after four days and seven days on all the CE membranes (*p* = 10^−4^; Figure 7). Among the four types of CE membranes, the 1% and 2% elastin membranes were significantly lower with the other membranes (*p* = 10^−4^).

In this study, the fibroblasts showed significant proliferation over seven days, suggesting the in vitro biocompatibility of the CE membranes. While not significant at every time point, there was a general trend for the 2% and 4% elastin-containing membranes to support greater fibroblast growths than the other membranes. Similar increases in cell growth were reported when elastin was added to other materials, such as PLGA, PCL, and collagen [17,18,19,20]. These results indicate that the incorporation has a beneficial effect on the cytocompatibility of the chitosan membranes. Improvements in the cytocompatibility may lead to enhancements in the ability of CE membranes to guide bone regeneration in vivo, which will be a focus of future studies.

## 4. Conclusions

This study presented the results on the electrospinning of a chitosan–elastin solution to increase the mechanical properties of chitosan-based GBR membranes. The chitosan membranes with elastin exhibited thicker fiber diameters, greater hydrophilicity, higher degradation rates, and higher mechanical strengths than the chitosan membrane. The FTIR spectra and the protein concentration test both proved that CE membranes with more elastin amounts showed higher elastin/protein concentrations, which lead to increased tear strengths and toughness of the membranes. Additionally, it showed the lack of cytotoxicity of the materials with elastin, suggesting potential enhancements in the membrane biocompatibility that could lead to improved guide bone regeneration. In conclusion, this study demonstrated that adding elastin to the electrospinning of chitosan effectively increases the tear strength and cytocompatibility of the membranes that might be useful for increasing their clinical handling ability and potential in guiding bone regeneration.

## Data Availability

Data are contained within the article.
